# Analysis of AI Precision Education Strategy for Small Private Online Courses

**DOI:** 10.3389/fpsyg.2021.749629

**Published:** 2021-11-10

**Authors:** Yu-Shan Lin, Ying-Hsun Lai

**Affiliations:** ^1^Department of Information Science and Management Systems, National Taitung University, Taitung, Taiwan; ^2^Department of Computer Science and Information Engineering, National Taitung University, Taitung, Taiwan

**Keywords:** small private online courses, O2O learning, precision education strategy, artificial intelligence, adaptive learning

## Abstract

In recent years, the learning efficacy of online to offline (O2O) teaching methods seems to outperform traditional teaching methods in the field of education. Students can use a small private online course (SPOC) teaching platform to preview class-related materials, learn basic knowledge, and enhance the practical experience of system development in offline courses. The research team applied an artificial intelligence (AI) precision education strategy to design a teaching experiment that evaluated whether this approach may lead to better learning outcomes. In addition to questionnaire surveys to ascertain students' attitudes toward and their satisfaction with learning, this study employed in-depth interviews to understand a potential influence on changes in teachers' curriculum design and teaching approaches when SPOCs was integrated into the traditional university classroom, as well as the impact of the AI precision education model. The results showed that the AI precision education model may facilitate students' learning experience and enhance student achievement.

## 1. Introduction

The rapid growth of massive open online courses (MOOCs) has resulted in novel teaching and learning methods. However, the following issues inevitably arise as a result of this global trend: (1) high registration rate and low completion rate; (2) a considerable gap in the interaction between students and teachers compared with traditional physical courses; (3) effective prevention of course certification and online evaluation still needs to be resolved; and (4) the cost of platform maintenance.

In recent years, models have begun to migrate from MOOCs to small private online courses (SPOCs). In a review titled “Forget MOOCs,” Oremus (2013) stated that free online courses should not replace teachers and classrooms but improve them. Professor Fox from the University of California, Berkeley, proposed SPOCs in 2013 to address the low completion rate of MOOCs (Fox, 2013). Contrary to MOOCs, this model advocates the use of MOOCs to supplement rather than substitute classroom teaching. They can improve teachers' teaching and learning. This method is also called “hybrid” or “hybrid learning” (Oremus, 2013). SPOCs have a high-class completion rate and solve the integrity issue associated with MOOCs' online evaluation through physical classroom examinations. In addition, because of the norms of class restrictions, it is easy to focus on individual students. Teachers can tweak the difficulty level of the course at any time. Incorporating flipped teaching in the course can be quite helpful to students' learning effectiveness (Goral, 2013). However, SPOCs also have shortcomings. For example, class restrictions deprive students of the right to choose specific courses; flipped teaching is integrated into the course requiring the instructor to commit additional time and energy. In addition, unlike MOOCs, SPOCs place a greater emphasis on the experience of course learners.

With time, the focus on differences in learners' traits continues to expand, from differences in explicit responses and learning outcomes to differences in mental organization and representation, cognitive styles, motivations, as well as an increased emphasis on differences in brain structure and learning-related issues. The perception of learners' differences in traits has gradually shifted from defects or specialties to unique and diversity. This change represents respect for each learner's differences and highlights the importance of education to assist learners in discovering and displaying individual strengths and increasing their chances of success (Pham et al., 2012). While teachers are frequently confronted with students' individual differences in the classroom, they can accommodate these differences through effective planning and related measures (Rosenfeld* and Rosenfeld, 2004).

Adaptive teaching has been re-conceptualized as a result of the diversified development of society. The early differentiation (ability grouping, sub-track learning) has evolved into the modern emphasis on the flexibility and diversity of students' learning styles. It is crucial to approach students' individual differences with a more positive attitude and pay attention to learning opportunities. This is the core meaning of adaptive teaching (Westwood, 2018). In flipped classrooms, students are expected to take ownership of their learning. However, there are still many students who are accustomed to relying on teachers and teacher-centered methods and struggle to be accountable for their learning. Therefore, instructors should pay special attention to individual differences among students while implementing flipped classrooms (Hao, 2016).

This personalized user requirement demonstrates the importance of integrating technical resources with the curriculum and the learners' actual circumstances. In recent years, the concept of “precision” has been extensively promoted in a variety of disciplines, with massive funds invested in related research and policy formulation. This fast-developing precision trend has inspired this research, which seeks to explore whether artificial intelligence (AI) assisted learning systems that intervene in a hybrid learning model can more effectively improve teachers' teaching and students' learning.

This study aims to compare whether the students have significant differences in learning effectiveness when using the online teaching course of MOOCs in conjunction with an offline teaching experiment platform (offline course) to conduct teaching experiments and introduce the intervention model of AI precision education. In addition, the study aims to gain a deeper understanding of the effectiveness of developing emerging technologies for innovative teaching. This study intends to use quantitative and qualitative data and teach experimental courses on the ShareCourse platform. It discusses innovative teaching strategies to compare students' learning effectiveness in the information departments of two national universities in the east.

The goal is to create high-quality MOOCs online in an offline learning environment to compare MOOC learners' online learning behavior and learning effectiveness using the same core course of information network to be jointly studied on the ShareCourse MOOCs platform. Following the course design and production, promoting the innovation and research of deep learning entails the following: first, exploring the online learning platform of MOOCs and integrating the challenges and difficulties of MOOC curriculum teaching; second, developing and redesigning MOOCs and integrating SPOCs; third, integrating the auxiliary teaching model of AI precision education; and fourth, promoting and applying the results of the learning effectiveness of MOOC teaching. Finally, we plan to integrate and analyze the teaching mode, potential, and effectiveness of MOOCs' multiple innovations based on research feedback and improvement.

Section 2 provides a review of the literature. Section 3 introduces the research methodology. Section 4 presents the results. Section 5 concludes the study and presents a discussion.

## 2. Literature Review

### 2.1. MOOCs and SPOCs

MOOC is an open digital learning method that enables a large number of learners to participate in the course via the web. The format differs from MIT's “Open Course Ware” (OCW), which began in 2002. The OCW provides a long-term summary of physical teaching. The course content is extensive, and the learner's attention span is short. In addition, there is a lack of interaction between learners. Scholars' opinions on MOOC quality can be separated into two categories: evaluations based on user benefits (Ehlers, 2004; Conole, 2014; Gamage et al., 2015) and assessments of the course development process (Yousef et al., 2014; Gan, 2018). Conole (2015) argued that the quality of MOOC courses should be measured from the user's perspective and proposed a 7C learning design framework, including the convergence and execution of curriculum vision, curriculum activity design, and overall design. Jang (2008) compiled the opinions of related scholars on the development process to evaluate the quality of MOOCs. He identified five measurement indicators for the teaching team: teaching content, teaching effectiveness, teaching resources, and teaching technology. Williams et al. (2012) proposed standard practices of curriculum syllabus design, curriculum design (including teaching materials and production design, open educational resource application, evaluation design), curriculum provision (including technical infrastructure, virtual teaching environment), technical support, resource input, teacher incentives, and student support.

Learning analysis and evaluation are essential factors in the design of MOOCs. Learning analysis can assist in mastering the learning process by identifying learners' challenges and preferred learning methods. Evaluation enables learners to understand solutions from teaching videos or course content with explanations, learning effectiveness, and different evaluation methods (Yousef et al., 2014). MOOC instructional videos play an essential role in the curriculum. MOOCs require appropriate video content design and shooting methods. Concise videos must attract learners to continue learning (Shoufan, 2019). Additionally, the videos should be of high quality, including video and audio clarity, lighting and image synchronization, and should be relevant to the course content and teaching evaluation (Hibbert, 2014).

SPOCs are open-course formats derived from MOOCs. Small refers to a small number of students (the number is restricted to a few hundred), and private refers to the students enrolled in the course at the school where the course has started, or the number of students is limited according to the course's characteristics. The term “online students” refers to courses that are not open to everyone on a large scale, intending to enhance learning participation and the course completion rate (Laaser, 2015; Xu et al., 2019). The SPOCs are characterized by their ability to improve teaching effectiveness (Wang et al., 2016). The teaching mode of SPOCs is based on the high-quality video content of MOOCs, which students can utilize to gain a basic understanding of a specific topic before class. As a result, teachers can teach higher-level content, respond to students' questions, or provide other exercises and additional learning materials in physical classrooms to provide a more comprehensive learning experience. Therefore, SPOCs are taught using a combination of face-to-face classroom instructions and online self-study. This is in contrast to the online format of MOOCs. In other words, SPOCs are MOOCs combined with classroom instructions. They are flipped hybrid courses that effectively use MOOC resources in classroom teaching (Ak and Gülseçen, 2018). SPOCs differ from MOOCs, which are available to an unlimited number of students. Therefore, SPOC students can communicate with teachers, receive high-quality teaching, and participate in course-related activities. If MOOCs are compared to sports equipment, SPOCs are similar to personal sports coaches, providing learners with additional guidance (Kaplan, 2017; Ruiz-Palmero et al., 2020).

### 2.2. AI Precision Education

With the rapid advancements in AI, it is necessary to take the initiative to transform the “teacher-oriented” education model into a “student-oriented” intelligent education model, as well as to construct a parallel teaching technology system that is interactive between virtual and physical reality (Tuomi et al., 2018; Pedro et al., 2019). Students, being self-directed learners, have varying abilities for learning and knowledge acquisition. Therefore, their strengths and weaknesses in subjects vary; the time required for learning and concentration, and the adaptation to the learning rhythm also vary.

In the traditional education model, all students use the same learning materials and follow the same learning rhythm, thereby concealing students' individual differences and preventing them from achieving the highest level of learning effectiveness. Education should be individualized and differentiated (McTighe and Brown, 2005). In addition to general education, students must be taught to follow their aptitudes. Information education, in particular, makes use of rapidly developing and mature technology to automatically push one-to-one teaching. The realization of precise education tailored to the unique characteristics of different students will be a new model of education in the future (Duong et al., 2019; Luan and Tsai, 2021).

In recent years, the wave of AI has swept the world. AI, in particular, can make personalized and accurate predictions, creating a type of precision education. AI is a technological science concerned with studying and developing theories, methods, technologies, and application systems for simulating, extending, and expanding human intelligence. Precision education is an educational philosophy and behavior based on big data analysis (Makhluf, 2020; Tsai et al., 2020). The first essential aspect of precision education is identifying and acknowledging differences, as well as providing targeted education and training according to the characteristics of individual students so that they can develop their personalities and coordinate their development. Precision education is based on big data technology, which is classified according to the different characteristics of students during the learning process to provide targeted education, develop their strengths or compensate for their weaknesses, and improve students' acceptance of knowledge through effective teaching behavior (Ellis, 2019).

## 3. Methodology

This study conducted teaching experiments using the online course of National Tsinghua University's MOOCs online learning platform and an offline course of the SPOC small-scale flipped classrooms of National Taitung University and National Dong Hwa University. The core knowledge and teaching content of the courses at the two schools were nearly identical. This new teaching method aims to improve students' learning effectiveness and allows them to combine theory with practice in order to enhance the quality of teaching and close the gap between learning and application.

**Figure 1:**
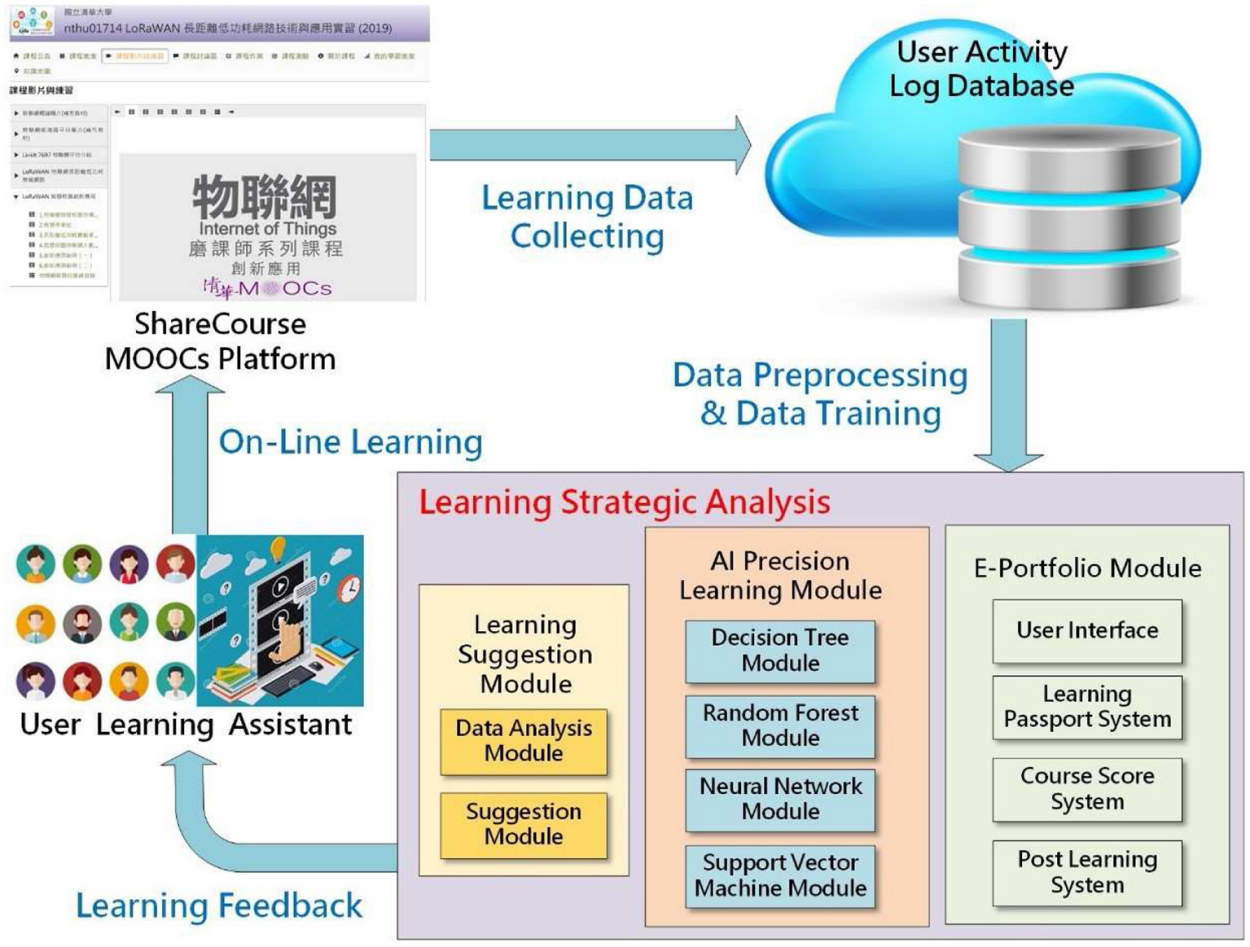
AI precision education assisted learning teaching model.

This study collected data using questionnaire surveys and systematically investigated and analyzed different research objects in the two schools. This study also used in-depth interviews to understand the two schools' different teaching modes and learning styles. These interviews also helped understand changes in teachers' curriculum design and teaching design as a result of the integration of MOOCs into the university classroom, as well as the impact of the AI precision education assisted learning teaching model shown in Figure 1, on students' attitudes, learning behaviors, and learning satisfaction.

### 3.1. Research Objects

The research participants of this study were students from Taitung University and Dong Hwa University who participated in the SPOCs mixed learning course. The sample sizes for Taitung University and Dong Hwa University were 42 and 21, respectively. Students from Dong Hwa University served as the experimental group, whereas students from Taitung University served as the control group, implying that AI precision education was only conducted at Dong Hwa University. The method was then assessed to see whether it could effectively improve students' learning effectiveness.

### 3.2. Research Design and Process

In addition to conducting questionnaire surveys to understand the impact of different AI precision education teaching models on student learning effectiveness, in-depth interviews were used to understand the changes that different teaching models have brought to teachers and teaching assistants in curriculum and teaching design. To analyze data from the student login data of the course platform is to understand the impact of different students' participation enthusiasm on students' learning effectiveness and learning satisfaction.

Before the experiment, all participating students from the two schools took the “Internet Information Knowledge Basic Ability Test” to ascertain their baseline knowledge. Next, a *t*-test was performed to check whether there was a significant difference in the levels of students from the two schools. While the course is in progress, teachers can design and adjust their teaching activities to meet students' specific needs. The platform provides course videos, handouts, exercises, discussion spaces, teaching assistant responses, and learning analysis graphs to aid students' self-directed learning. This course is structured into four major stages: an introduction to the ShareCourse platform, basic capabilities of the Internet of Things, O2O hybrid teaching, and learning assessment.

The “Course Learning Experience Questionnaire” was distributed prior to the conclusion of the course. This questionnaire was used to understand students' attitudes and learning-related issues. In addition, in-depth interviews were conducted with course teachers and teaching assistants to learn more about their perspectives on the SPOC teaching mode and AI precision education intervention (experimental group). Finally, a learning achievement test was conducted to assist teachers and students in understanding the learning effectiveness. Furthermore, it was determined whether a substantial difference in students' performance between the two schools exists to understand whether the AI-assisted learning system can truly improve learning effectiveness.

### 3.3. Research Tools

**Figure 2:**
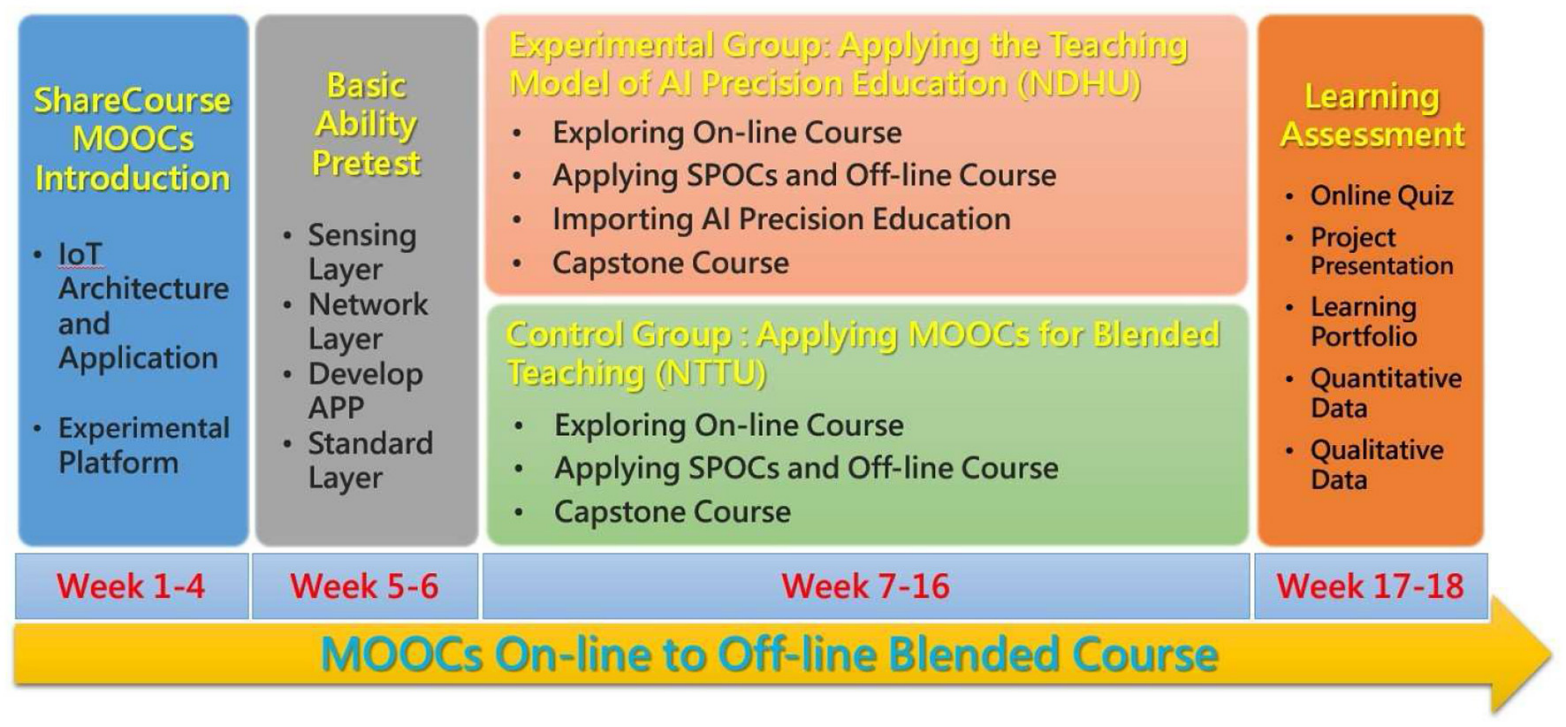
Course experiment structure.

The learners registered in courses on the ShareCourse learning platform were subjected to a field experiment in this study. The two schools collaborated on a ten-week MOOC online learning course and a blended learning course using MOOCs and SPOCs. At Dong Hwa University, AI precision education was introduced to explore precision education. Figure 2 depicts the structure of the course experiment and the effect of various teaching modes of intervention on students' learning effectiveness. The field experiment method involves manipulating one or more independent variables (experimental variables) in a real-world environment while maintaining as much control as possible. Due to the fact that field experiment is conducted in the field and manipulated in a real-world setting, items and irrelevant control variables can more accurately reflect the actual situation.

Students' learning effectiveness was evaluated based on academic achievement tests and learning satisfaction (measured using the self-reporting scale). The former's measurement index is based on the learning goals planned by the curriculum, and relevant questions are included in online examinations to assess learners' learning achievements. The latter measure is based on the course learning experience questionnaire, with the following description: the questionnaire used SPSS statistical software to analyze the quantitative data. In addition to the quantitative research discussed above, the qualitative research method involving in-depth interviews was employed to understand better teachers' and teaching assistants' perspectives on this innovative teaching model. Additionally, innovative teaching methods and effectiveness evaluations of AI precision education have been introduced for offline courses.

#### 3.3.1. Course Learning Experience Questionnaire

This course learning experience questionnaire was primarily designed to understand students' views on their learning experiences, as well as the impact of AI precision education intervention (experimental group) on learning. This questionnaire is divided into eight sections: learning satisfaction (including the teacher's role and teacher-student interaction as perceived by students), readiness for online learning, self-study evaluation, learner/teacher positivity, learning activities, comprehensive issues (such as the quality of teaching videos, the quality of online and offline interactions, and the suitability of SPOC teaching models), basic personal information, and other suggestions. The experimental group had one additional part: views on AI precision education intervention.

#### 3.3.2. AI Precision Education

The AI precision education-assisted learning system collects the MOOC platform's relevant learning history data records. The data set contains all of the learner's learning history information (the number of course materials downloaded, the number of views on the video, the number of video downloads, and so on) and is used as a training data set to produce a set of precision-learning auxiliary modules. The AI precision education-assisted learning system typically uses four classification learning methods: decision tree, random forest, neural network, and support vector machine modules. Due to the sample size limitation, this study relied exclusively on the support vector machine (SVM) method to evaluate and classify learners' learning history data records and predict learners' online learning effectiveness. The training sample set for this study was the relevant learning data recorded during the mid-term exam, and the mid-term results were used to evaluate the student's learning effectiveness. The mid-term test comprised the relevant test questions listed separately according to the course's online learning units, which included basic concepts of learning, advanced tests, and extension tests. Following model training, the relevant model was used to predict whether students would achieve the learning effectiveness of each subsequent online learning.

Subsequently, teachers provide special guidance for students in need to improve learning effectiveness and achieve the final results of AI-assisted precision education.

#### 3.3.3. In-depth Interview

In-depth interviews were conducted at this stage using semi-structured interviews. The study employed more open interview methods to gain insights into teachers' and teaching assistants' perspectives on the SPOC teaching mode and AI precision education intervention (experimental group). The interview process was recorded after the interviewee's consent was obtained to record the content of the interviewee's response. The outline of the interviews is as follows:

1. Use of MOOC platform and videos for teachers and teaching assistants.
2. Curriculum planning and design by teachers and teaching assistants.
3. Observation of changes in student learning (participation, motivation, effectiveness, and special events).
4. Attitudes toward AI precision education intervention (experimental group).
5. Future improvements.

## 4. Results

This study aims to determine the impact of different teaching modes of AI precision education on students' learning effectiveness. The remainder of this section is divided into four parts. The first part uses questionnaire survey results to understand students' views on the learning experience, including classroom learning satisfaction and learning effectiveness. The second part evaluates the learning effectiveness of each student using the AI precision education-assisted learning system. The third part compiles the results of the in-depth interviews, and the last part presents the results of the statistical verification of the students' performance of the two schools before and after the experiment.

### 4.1. Curriculum Learning Experience Questionnaire Survey Results

The average of students' responses to questions about learning satisfaction, learner/teacher positivity, learning activities, and comprehensive problems from both schools was higher than 4 (Table 1), indicating that students generally agree with the effectiveness of O2O teaching methods. This is because students can preview the teaching content using the MOOC teaching platform before class. When faced with an issue, students seek assistance from a variety of sources, such as by asking the teacher or searching online for solutions. Simultaneously, students believe that offline experimental courses can impart essential knowledge and experience necessary for system development. Nevertheless, the average in the self-study evaluation part of Dong Hwa University was low, indicating that most students lacked learning confidence. This is because two-thirds of the students are majoring in the Information Management Department of the School of Management, not Information Engineering, and thus have lower learning confidence than students in the professional field. However, the majority of them agree that teachers can adjust the teaching content flexibly to accommodate individual differences among students, so it does not make much difference in the overall learning effect. The survey results from Taitung University show that online learning readiness and self-study evaluation averages are higher. One probable explanation for this is that all of Taitung University's elective students are from the Department of Information Engineering. Their mastery, familiarity, and interest in professional information courses are relatively high, which translates into a relatively high degree of agreement on these items.

**Table 1:** Average of curriculum learning experience questionnaire survey.

**Items**	**NDHU**	**NTTU**
**(1) Learning satisfaction**		
1. I think the learning effect is better than the general physical class.	4.24	4.1
2. Teacher-student interaction is good in the courses taught, and guide learning is created.	4.62	4.12
3. Teachers respond appropriately and constructively to students' questions or assignments.	4.52	4.07
4. The teacher's teaching attitude is enthusiastic and serious.	4.57	4.14
5. Teachers express clearly, explain, and demonstrate clearly.	4.48	4.12
6. This course enhances my ability in this field.	4.19	4.24
7. I am willing to continue to take other online and physical combination courses.	4.24	4.19
8. I would like to recommend others to take online and physical combination courses.	4.33	4.19
9. Overall, I am satisfied with the experience of this course.	4.52	4.14
**(2) Readiness for online learning**		
1. I can plan online time, location, and learning tools according to the weekly learning progress requirements arranged by the teacher.	3.86	4.17
2. I can effectively use the time to watch teaching videos before class to achieve the learning progress specified by the teacher.	3.76	4.12
3. I can connect the new concepts learned by watching the instructional video before class with the learning tasks arranged by the teacher in the class.	4.05	4.21
4. When I encounter a problem in watching the instructional video specified by the teacher before class, I can try to ask for help (for example, find the answer on the Internet).	4.1	4.36
**(3) Self-study evaluation**		
1. I think I have a good learning attitude.	3.57	3.95
2. I think I have a good understanding of the concepts of this course.	3.71	4.02
3. I have a high willingness to learn.	3.81	4.12
4. I have a high degree of confidence in learning.	3.86	4.1
**(4) Learner/teacher positivity**		
1. In general, the students are actively involved in this course.	4.29	4.26
2. The teacher actively participates in the course.	4.62	4.1
3. I think the teacher can flexibly adjust the teaching content according to the individual differences of students.	4.33	4.07
**(5) Learning activities**		
1. I can accept the learning mode of hands-on practice and interactive discussion in class.	4.48	4.36
2. The practical activities of the offline experimental courses let me learn the basics of the Internet.	4.29	4.21
3. The practical activities of offline experimental courses let me learn system development experience.	4.29	4.45
**(6) Comprehensive issues**		
1. I think the teaching videos are of good quality.	4.38	4.29
2. I think the quality of online and offline interaction is good.	4.24	4.17
3. I think the teaching mode combining online and physical courses is suitable.	4.48	4.14

An independent samples *t*-test was performed to understand whether there is a significant difference in the average of students between the two schools. There were no significant effects on learning satisfaction [*t*(61) = 1.329, *p* = 0.189], readiness for online learning [*t*(61) = −1.405, *p* = 0.165], self-study evaluation [*t*(61) = −1.584, *p* = 0.118], learner/teacher positivity [*t*(61) = 1.357, *p* = 0.180], learning activities [*t*(61) = 0.044, *p* = 0.965], or comprehensive issues [*t*(61) = 0.867, *p* = 0.389]. However, when the question was tested one by one, it was found that there were significant differences in item 2 [*t*(61) = 2.333, *p* = 0.023] and item 3 [*t*(61) = 2.039, *p* = 0.046] of learning satisfaction, as well as item 2 [*t*(61) = 2.196, *p* = 0.032] of learner/teacher positivity. This shows that the means of Dong Hwa University in these items are significantly higher than those of Taitung University. This demonstrates that the AI-assisted learning system's intervention affects students' perception of teacher-student interaction, teachers' appropriate and constructive responses to students, and teachers' active participation in the course.

### 4.2. Results of AI Precision Education

Regarding the AI precision education-assisted learning system, the study used MOOCs to teach students basic theoretical knowledge and for offline implementation of low-power long-distance wireless networks. Thus, determining whether a student's learning effect is good and whether additional guidance is needed to achieve a certain degree of learning effectiveness is critical. The study used SVM as the model to make judgments to identify the students' learning situation and consider ways to efficiently reduce errors when dealing with low data volume. To complete the training of the SVM model, it is necessary first to determine which features need to be imported into the SVM. Because MOOCs can fully record students' learning processes, including the number of times a video is watched, viewing time, practice time, and online test results, the indicators above are incorporated as characteristic values. Online test results were utilized to determine the learning outcomes. The number of times a video was viewed, viewing time, and exercises were integrated and set as learning parameters. The study defines *o*_*w*_ as the number of times a video is viewed, *o*_*t*_ as the viewing time, and *o*_*p*_ as the number of exercises, and assigns a weight to each parameter. Therefore, the following are the overall characteristic parameters:


(1) $$\begin{array}{r} x_{i}=w_{1}o_{w}+w_{2}o_{t}+w_{3}o_{p} \end{array}$$

**Figure 3:**
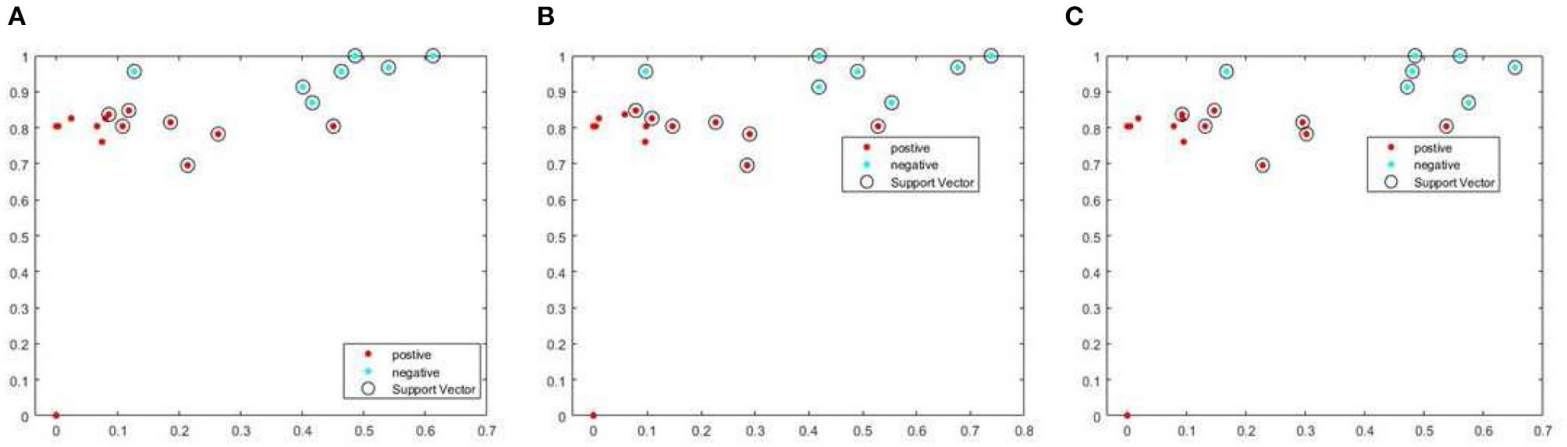
The impact of different weights on AI precision education. **(A)** 0.18:0.4:0.42, **(B)** 0.19:0.13:0.68, and **(C)** 0.37:0.26:0.37.

Next, this study used basic ability test scores as an additional feature to assist the SVM in determining students' learning effectiveness. After successfully categorizing students' learning effectiveness, this approach provides personalized tutoring for students in need, enabling them to develop the confidence necessary to improve their learning effectiveness. Finally, this study evaluates the effectiveness of the AI precision education-assisted learning system using the learning achievement test as an indicator. The results of the model trained using SVM are shown in Figure 3. The use of an SVM can effectively classify students' learning status. Because this study required data collection in the classroom, the overall data volume is insufficient because of the number of students in the course. However, the accuracy of the trained model has surpassed 70%, indicating that it has a certain degree of reference. In addition, Figure 3 illustrates the effect of assigning different weights to the training results. This shows that the longer the viewing time and the greater the number of views, the more precisely the overall accuracy can be improved for learning effectiveness. In other words, because MOOCs enable students to learn before and after class, their understanding of basic theoretical knowledge improves, which has an impact on their overall academic performance.

### 4.3. In-depth Interview Results

The most important aspects of using the MOOC platform and videos are the course progress, course videos, after-school exercises, classroom tests, and course discussion areas. In addition, the on-campus learning platforms, namely Dong Hwa's e-Learning and Taitung University's eClass, are used simultaneously. The course planning and design part is mainly O2O. Online videos allow students to grasp the concepts clearly before moving on to the implementation, and the connection is seamless. In addition, because the course is required to produce thematic results at the conclusion of the semester, it is based on network programming and the concept of Long Range (LoRa). Related theories and practical content are planned according to the student's topic. For example, concepts employed in related topics such as automatic watering, temperature sensing, mobile phone recording, and transmission will be discussed, similar to the ideas of micro-credits and micro-courses.

Regarding the observation of student learning changes at Dong Hwa University, the learning materials on the platform are vital for students with non-professional backgrounds (Information Management Department) at the beginning of the course. They watched the video on the platform more seriously. Furthermore, due to their familiarity with the basic concepts of networking and the ability to develop programs, students with a professional background in information technology (Information Engineering) believed that they already understood all the conceptual explanation videos; hence, they watched less videos in comparison. The advantage of MOOCs is that they can assist in learning. Students who are motivated to learn can access a wealth of knowledge on the platform. At Taitung University, students watched the teaching videos on the platform before class, which resulted in increased discussion in the class, and they could understand the content explained by the teacher more quickly. On the whole, compared to physical courses, this learning platform collects and analyzes the learning materials of MOOC learners to ascertain their learning conditions, difficulties, and learning modes and adjusts the teaching content and methods accordingly. As a result, students' learning motivation and participation, as well as the learning effect, have significantly improved.

Both teachers and teaching assistants agreed with this approach regarding the attitude of AI precision education intervention. Special counseling can be provided for learners with poor learning conditions based on the students' learning history, through the AI system grouping, to identify problems early and further improve learning effectiveness, successfully achieving AI precision education-assisted learning, and teaching students according to their aptitude.

### 4.4. *T*-Test Result of Basic Ability Test and Learning Achievement Test

Independent samples t-tests were used to determine whether there was a significant difference in the scores of the students from the two schools on the basic ability test of information knowledge before the experiment. In Table 2, there was no significant difference in the scores of the students from the two schools before the experiment [*t*(61) = 0.868, *p* = 0.389]. However, there was a significant difference in the learning achievement tests of the two schools' students after the experiment [*t*(61) = 2.627, *p* = 0.011]. This demonstrates how the AI-assisted learning system can improve students' learning effectiveness.

**Table 2:** Summary of *t*-test results of the two school students' score before and after the experiment.

	**University**	**Sample size (*n*)**	**Mean (M)**	**Standard deviation (SD)**	**Degree of freedom (df)**	***t*-value (t)**	***P*-value (p)**
Basic	NDHU	21	64.524	9.9881	61	0.868	0.389
ability test	NTTU	42	61.643	13.4524			
Learning	NDHU	21	80.143	6.3819	61	2.627	0.011^*^
achievement test	NTTU	42	75.548	6.6230			

* *p < 0.05*.

## 5. Discussion

Online teaching videos enable students to understand the basic concepts of the course before taking the physical course, and the connection can be smoother in the physical course. At the beginning of the course, the learning materials on the platform were vital for students with a non-professional background (Information Management Department). They watched the teaching videos on the platform more seriously. In fact, the advantage of MOOCs is that they are flexible, allowing students to learn at their own pace without being restricted by time and space, which assists learning, as Fellman et al. (2020) also indicate in their research. Students who are determined to learn can accomplish a great deal on the platform. Furthermore, because students watched the teaching videos before class, the discussion in the class and the interaction between teachers and students increased, allowing students to learn more and teachers to teach more content. This is an example of a virtuous circle. Because information courses emphasize students' hands-on operations, offline thematic practice enables students to apply the acquired knowledge to practical problem-solving.

Although the test results of the two schools' course learning experience questionnaire surveys were not significant, the achievement test scores of the two schools' students following the experiment revealed a significant difference. There was no difference in the initial basic ability test scores prior to the experiment. However, there was a significant difference in the achievement test scores after the experiment, indicating that the AI precision education-assisted learning system can effectively assist students in learning. As Dekker et al. (2020) concluded, AI intervention increases students' academic performance. The purpose of AI precision education-assisted learning can be successfully achieved by utilizing students' learning history data, the AI system grouping, special counseling for learners with poor learning conditions, early detection of problems, and intervention to further improve learning effectiveness. This is consistent with the study of Lu et al. (2018), which found that precision education aims to identify high-risk students as early as possible and intervene in a timely manner based on teaching experience. Similarly, Luan et al. (2020) stated that AI has the potential to realize individualized learning and precision education. This is because a smart education system powered by AI technology is capable of collecting precise and comprehensive personal data, which can then be analyzed to reveal students' learning patterns and determine their specific needs. Since teachers are aware of their students' specific needs, they can provide timely assistance to further improve students' learning effectiveness.

The research limitation was the smaller number of samples. This is because the sample size was restricted to students enrolled in the courses. Increased sample size can improve the accuracy of the model trained by AI and enhance the interpretation of the results. Future studies should consider large-scale research and see whether any other variables influence students' learning experiences. Additional AI training models, such as decision trees, random forests, and neural networks, can also supplement the research. In addition, experimenting with the same courses taught by the same teacher in different classes or the same course offered in different semesters may result in less interruption during the experiment process.

## Data Availability Statement

The original contributions presented in the study are included in the article/Supplementary Material, further inquiries can be directed to the corresponding author/s.

## Author Contributions

Y-SL proposed the whole research theory, conducted literature research, and designed course experiment flow of AI precision education. Y-HL collected experimental data and analyzed the data. All authors contributed to the article and approved the submitted version.

## Conflict of Interest

The authors declare that the research was conducted in the absence of any commercial or financial relationships that could be construed as a potential conflict of interest.

## Publisher's Note

All claims expressed in this article are solely those of the authors and do not necessarily represent those of their affiliated organizations, or those of the publisher, the editors and the reviewers. Any product that may be evaluated in this article, or claim that may be made by its manufacturer, is not guaranteed or endorsed by the publisher.
